# Safety and pharmacokinetics of recombinant human hepatocyte growth factor (rh-HGF) in patients with fulminant hepatitis: a phase I/II clinical trial, following preclinical studies to ensure safety

**DOI:** 10.1186/1479-5876-9-55

**Published:** 2011-05-08

**Authors:** Akio Ido, Akihiro Moriuchi, Masatsugu Numata, Toshinori Murayama, Satoshi Teramukai, Hiroyuki Marusawa, Naohisa Yamaji, Hitoshi Setoyama, Il-Deok Kim, Tsutomu Chiba, Shuji Higuchi, Masayuki Yokode, Masanori Fukushima, Akira Shimizu, Hirohito Tsubouchi

**Affiliations:** 1 HGF Hepatic Regeneration Therapy Project, Department of Experimental Therapeutics, Translational Research Center, Kyoto University Hospital, Kyoto, Japan; 2 Digestive Disease and Life-style Related Disease, Kagoshima University Graduate School of Medical and Dental Sciences, Kagoshima, Japan; 3 Department of Clinical Innovative Medicine, Translational Research Center, Kyoto University Hospital, Kyoto, Japan; 4 Department of Clinical Trial Design and Management, Translational Research Center, Kyoto University Hospital, Kyoto, Japan; 5 Department of Gastroenterology and Hepatology, Kyoto University Graduate School of Medicine, Kyoto, Japan; 6 R&D and Corporate Integration, Kyoto University Graduate School of Medicine, Kyoto, Japan; 7 Department of Experimental Therapeutics, Translational Research Center, Kyoto University Hospital, Kyoto, Japan

## Abstract

**Background:**

Hepatocyte growth factor (HGF) stimulates hepatocyte proliferation, and also acts as an anti-apoptotic factor. Therefore, HGF is a potential therapeutic agent for treatment of fatal liver diseases. We performed a translational medicine protocol with recombinant human HGF (rh-HGF), including a phase I/II study of patients with fulminant hepatitis (FH) or late-onset hepatic failure (LOHF), in order to examine the safety, pharmacokinetics, and clinical efficacy of this molecule.

**Methods:**

Potential adverse effects identified through preclinical safety tests with rh-HGF include a decrease in blood pressure (BP) and an increase in urinary excretion of albumin. Therefore, we further investigated the effect of rh-HGF on circulatory status and renal toxicity in preclinical animal studies. In a clinical trial, 20 patients with FH or LOHF were evaluated for participation in this clinical trial, and four patients were enrolled. Subjects received rh-HGF (0.6 mg/m^2^/day) intravenously for 12 to 14 days.

**Results:**

We established an infusion method to avoid rapid BP reduction in miniature swine, and confirmed reversibility of renal toxicity in rats. Although administration of rh-HGF moderately decreased BP in the participating subjects, this BP reduction did not require cessation of rh-HGF or any vasopressor therapy; BP returned to resting levels after the completion of rh-HGF infusion. Repeated doses of rh-HGF did not induce renal toxicity, and severe adverse events were not observed. Two patients survived, however, there was no evidence that rh-HGF was effective for the treatment of FH or LOHF.

**Conclusions:**

Intravenous rh-HGF at a dose of 0.6 mg/m^2 ^was well tolerated in patients with FH or LOHF; therefore, it is desirable to conduct further investigations to determine the efficacy of rh-HGF at an increased dose.

## Background

Acute liver failure (ALF) is a rare but fatal clinical syndrome marked by the abrupt loss of hepatic cellular function, with the subsequent development of coagulopathy, jaundice and encephalopathy [1-3]. In Japan, ALF with the histological appearance of hepatitis, caused by viral infection, autoimmune hepatitis and drug allergy-induced liver injury, is classified as fulminant hepatitis (FH) or as the related disease late-onset hepatic failure (LOHF) [4]. FH is identified as hepatitis in which hepatic encephalopathy develops within 8 weeks after the onset of disease symptoms, with prothrombin time (PT) less than 40% of the standardized values. Also, FH is further classified into two subtypes: acute (FHA) and subacute type (FHSA) in which the encephalopathy occurs, respectively, within 10 days or after 11 days or more. Patients in whom the encephalopathy develops between 8 and 24 weeks after disease onset with PT less than 40% are diagnosed as having LOHF. This distinction is useful in guiding prognosis: the time to onset of encephalopathy is negatively correlated with outcome. The only effective therapy for FH is liver transplantation. Other therapies, including corticosteroids, have no demonstrable benefit [5], lamivudine for acute hepatitis B [6], and plasmapheresis [7]. Therefore, patients with FH who did not receive liver transplantation had extremely poor prognoses: the survival rates were 53.7% in FHA and 24.4% in FHSA, and 11.5% in LOHF in Japan [4].

Hepatocyte growth factor (HGF) was first purified as a potent mitogen for hepatocytes from the plasma of patients with FH [8,9]. HGF is one of the primary agents promoting the proliferation of mature hepatocytes [10-12]. The stimulatory effect of HGF on liver regeneration has been observed *in vivo *using normal and partially hepatectomized rats [11]. Additionally, HGF stimulates proliferation of hepatic progenitor cells, which appear following hepatic injury [13]. Furthermore, recent investigations using mice deficient in c-met, a specific receptor for HGF, demonstrated that the HGF/c-met signaling pathway is essential for efficient liver regeneration and repair [14,15]. Conversely, HGF exerts protective and anti-apoptotic functions toward hepatocytes *in vitro *[16-18] and *in vivo *[19-21], and is able to prevent Fas (CD95/APO-1)-triggered death of adult hepatocytes, leading to rescue from Fas-induced fulminant hepatic failure [20]. These results indicate that HGF has the potential to be a new therapeutic agent for ALF through its mitogenic and anti-apoptotic activities.

We have worked to develop translational medicine protocols for recombinant human HGF (rh-HGF), and have performed an investigator-initiated International Conference on Harmonization of Technical Requirements for Registration of Pharmaceuticals for Human Use (ICH)-Good Clinical Practice (GCP)-registered phase I/II clinical trial of rh-HGF. As this application is the first clinical trial to administer rh-HGF to humans, we performed additional preclinical studies to ensure minimization of the predicted side effects, and then treated four patients with repeated doses of rh-HGF in order to evaluate the safety, pharmacokinetics and clinical efficacy of FH therapy.

## Methods

### Animal experiments to ensure safety of rh-HGF administration

#### Animals

Female Crown miniature swine, six to seven months of age, and male Wistar rats, seven weeks of age, were obtained from Japan Farm (Kagoshima, Japan) and Charles River Laboratories Japan Inc. (Yokohama, Japan), respectively. The animals were maintained under constant room temperature (25°C), and given free access to water and the indicated diet throughout the study. The protocol for animal studies was approved by the ethics committee of the Graduate School of Medicine, Kyoto University (Kyoto, Japan). All animal experiments were performed after one to three weeks acclimation on a standard diet.

#### General pharmacological test

After Female Crown miniature swine were anesthetized by inhalation of sevoflurane, nitric dioxide and oxygen, catheters were inserted into one internal jugular vein (for injection of rh-HGF) and to one common carotid artery (to measure BP). One mg/kg of rh-HGF was injected through the internal jugular vein over the course of 20 min. HR was recorded by electrocardiographic monitoring, and cardiac function was measured via echocardiography. To evaluate the effect of stepwise infusion of rh-HGF on BP, 0.4 mg/kg of rh-HGF was injected over the course of three hours, with a stepwise increase in dose rate (10% of the total dose over the first 60 min, 30% over the next 60 min, and 60% over the last 60 min) through the catheter inserted into an internal jugular vein.

#### Evaluation of renal toxicity of repeat dose of rh-HGF

rh-HGF (0.4, 1.0 and 4.0 mg/kg) was administered to rats intravenously in a bolus for 14 days, followed by observation for 2 weeks. Urinary excretion of albumin and protein were measured periodically during and after rh-HGF administration. Animals were sacrificed at the ends of rh-HGF administration (day 14) and the observation period (day 28) to evaluate renal involvement, including serum creatinine and histological findings.

### A phase I/II clinical trial for patients with acute liver failure

#### Overview

This single-arm, open-labeled, and dose-escalation study was conducted at Kyoto University Hospital, Kyoto, Japan. Study protocols were reviewed and approved by the Investigational Review Board and Ethics Committee governing Kyoto University Hospital before the commencement of patient enrollment. Studies were performed in accordance with principles of GCP, and conformed to ethical guidelines of the Declaration of Helsinki. All participating patients, or (when participants were not able to subscribe because of hepatic encephalopathy) their legal representatives provided written informed consent before being enrolled into the study.

#### Selection of patients

Consenting patients were prospectively screened from September 2005 to June 2008. Eligible patients with FHSA or LOHF, who were not able to receive liver transplantation, met at least one of the following four parameters: (1) aged 45-year-old or above, (1) PT 10% or less of the standardized values, (3) total bilirubin (T-Bil) level of 18.0 mg/dL or more, or (4) direct/total bilirubin ratio less than 0.67. The following patients were not eligible: those under 16 years old; those treated with glucagon and insulin, or prostaglandin E1 48 hours before registration; those with presence or past-history of malignant tumors; those with heart failure; those with severe complication including pneumonia, sepsis, disseminated intravascular coagulation syndrome or gastrointestinal bleeding; and those with allergic reaction against rh-HGF. Pregnancy-aged women were also ineligible, because toxicity of rh-HGF to reproductive development in female animals has not been examined. Additionally, patients were also excluded on the grounds of renal involvement, including urinary excretion of ≥1 mg/mL protein, deformed red blood cells or RBC casts in sedimentary urine, a serum creatinine level of 2.0 mg/dL or more, or urine volume less than 400 mL/day.

#### Protocol therapy and observation after rh-HGF dosing period

rh-HGF was prepared as a GMP-grade material. The initial dose of rh-HGF was fixed at 0.6 mg/m^2^/day, which ensured not only safety but also clinical efficacy, as determined by several preclinical animal studies. In this dose escalation study, dose of rh-HGF can be increased from the initial dose (0.6 mg/m^2^) to 1.2, 1.8 or 2.4 mg/m^2^. rh-HGF was administered intravenously with a stepwise increase during 3 hours for up to 14 days, followed by a 14-day observation period. All patients were followed in order to determine the outcomes after the study period (up to 28 days).

#### End points

The primary endpoint of interest was the safety of repeated doses of intravenous rh-HGF, which was evaluated on the basis of the occurrence, frequency, and severity of adverse events. All patients were treated in an intensive care unit. During the on-study period, patients were monitored for safety at regular intervals from the start of rh-HGF administration until 14 days after completion of study drug dosing. Safety assessments included physical examination, clinical laboratory test and adverse events. Adverse events were monitored throughout the duration of the study, and evaluated in terms of adverse events graded according to the Common Toxicity Criteria grading system. Causal association of adverse events with rh-HGF was determined by clinician's best judgment. All adverse events were treated appropriately regardless of the cause; where necessary, patients were withdrawn from the study. The incidence of adverse events was computed from the number of patients experiencing at least one adverse event from among those who received at least a single dose of rh-HGF.

The secondary endpoints were the pharmacokinetics of intravenously injected rh-HGF and clinical efficacy, including survival period and outcome. To examine pharmacokinetics of rh-HGF, blood samples were collected for analysis of rh-HGF at multiple time points on days 1, 3, 5, 8, and 11 for assessment. Serum concentrations of HGF were determined by enzyme-linked immunosorbent assay (ELISA) (Otsuka Co., Ltd., Tokushima, Japan) [22]. Laboratory data, including PT-international normalized ratio (PT-INR), T-Bil, serum albumin, alanine aminotransferase (ALT), and α-fetoprotein (AFP), were examined before plasma exchange or rh-HGF administration.

#### Statistical analysis

To evaluate survival benefits by administration of rh-HGF, the stratified proportional hazards model was used for analyzing matched datasets. All statistical analyses were done using SAS version 9.1 (SAS Institute, Inc., Cary, NC).

## Results

### Establishment of rh-HGF dosing method to respond to a decrease in blood pressure in miniature swine

**Figure 1:**
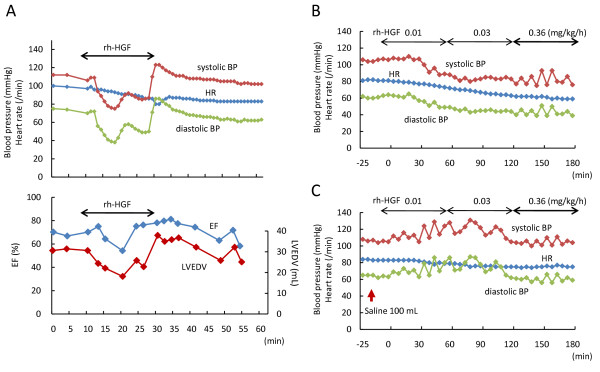
**Intravenous injection of rh-HGF reduced blood pressure through capacitance vessels in miniature swine**. Effect of intravenously administered rh-HGF on BP, HR, and cardiac function was examined in miniature swine under general anesthesia. (A) Intravenous injection of rh-HGF (1.0 mg/kg) rapidly reduced systolic and diastolic BP. Reduced BP was persistent during rh-HGF administration (for 20 min), and was immediately recovered after the rh-HGF injection. Echocardiography showed that ejection fraction (EF) and left ventricular end-diastolic volume (LVEDV) were reduced during rh-HGF administration. (B) rh-HGF (0.4 mg/kg) administered for three hours with a stepwise increase (0.01 mg/kg for first 60 min, 0.03 mg/kg for the next 60 min, and 0.36 mg/kg for the last 60 min) gradually decreased BP and HR. (C) Infusion of 100 mL of saline prior to rh-HGF administration prevented a decrease in BP during exposure to rh-HGF.

In general pharmacological tests, intravenous rh-HGF (1.0 or 0.2 mg/kg) caused a rapid decrease in systolic blood pressure (BP) in miniature swine, whereas respiratory status was not affected (data not shown). Therefore, before starting the clinical trial, we further investigated the effect of rh-HGF on circulatory status in miniature swine under general anesthesia. When a total dose of rh-HGF of 1.0 mg/kg was administered over the course of 20 min, a decrease in systolic BP occurred promptly, and continued throughout rh-HGF administration (Figure 1A). Although heart rate (HR) gradually decreased, no electrocardiographic abnormalities, including arrhythmia and ischemic changes, were observed throughout the experimental period. Additionally, cardiac ultrasonography showed a decrease in left ventricular end-diastolic volume (LVEDV) as well as ejection fraction (EF), in parallel with a decrease in BP, but no abnormalities of left ventricular movement (Figure 1A). These results indicate that intravenous injection of rh-HGF reduced BP through dilatation of capacitance vessels.

Next, we tried to develop a method for rh-HGF administration that would avoid rapid BP reduction. We finally established a stepwise infusion method in which rh-HGF was administered with a stepwise increase over the course of three hours (10% dose for 60 min, 30% for next 60 min, and 60% for the last 60 min) (Figure 1B). We found that appropriate infusion effectively prevented the decrease in BP caused by intravenous rh-HGF administration (Figure 1C). The preventive effect of additional infusion also supports the idea that dilatation of capacitance vessels is a cause of HGF-induced BP reduction.

### Evaluation of renal toxicity induced by repeated dose of rh-HGF in rats

**Figure 2:**
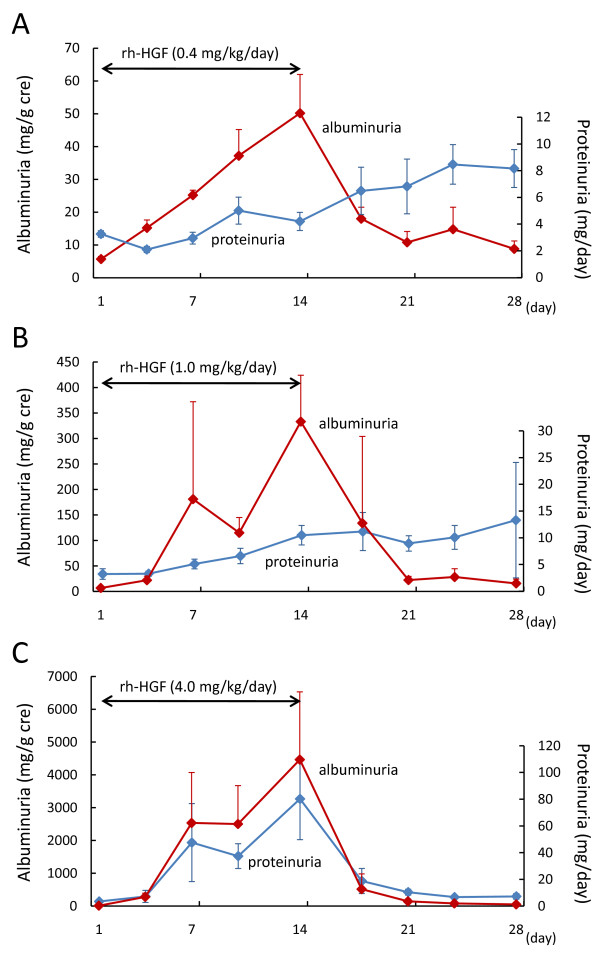
**Repeated dose of rh-HGF induced an increase in urinary excretion of albumin and protein in rats**. Rats were administered rh-HGF, 0.4 (A), 1.0 (B), and 4.0 mg/kg/day (C) (n = 4 for each), intravenously for 14 days, and urinary excretion of albumin and protein was measured before (day 1), during (days 7 and 14), and 7 and 14 days after HGF administration. Repeated doses of rh-HGF induced an increase in urinary albumin excretion in dose dependent manner. Urinary excretion of albumin was reversible even when dosing 4.0 mg/kg/day of rh-HGF (C), and preceded an increase in proteinuira in rats treated with 0.4 and 1.0 mg/kg of rh-HGF (A and B, respectively).

Repeated dose toxicity tests using rats or cynomolgus monkeys identified an increase in urinary excretion of albumin and protein as a potential adverse event in a clinical trial. Therefore, we further examined whether renal toxicity induced by repeated rh-HGF dosing for 14 days was reversible. We intravenously administered 0.4, 1.0, and 4.0 mg/kg/day of rh-HGF to rats for 14 days, followed by a 14-day observation. Urinary excretion of albumin increased in rats treated with rh-HGF from day 4 in a dose dependent manner (Figure 2). In animals treated with 0.4 or 1.0 mg/kg/day of rh-HGF, excretion of urinary albumin preceded an increase in proteinuria (Figure 2A and 2B). Conversely, neither serum creatinine nor BUN were affected throughout the experimental period, and increased urinary excretion of albumin gradually decreased after the completion of rh-HGF dosing during the 14-day observation period. In histological analysis, mesangial expansion, hyaline droplet deposition in glomeruli and tubules, and renal hypertrophy were observed after repeated doses of rh-HGF for 14 days; however, these histological findings were in the slight-to-mild range, and still identified as reversible changes (data not shown). In a clinical trial, the clinical dose of rh-HGF, 0.6 mg/m^2^, corresponds to 0.1 mg/kg in rodents. Therefore, renal toxicity, induced by repeated rh-HGF dosing for 14 days, would be predicted to be reversible; furthermore, excretion of urinary albumin is a useful way to monitor renal toxicity.

### Patient characteristics

Between September 2005 and June 2008, 20 patients with FHSA or LOHF were evaluated for participation in the clinical trial of rh-HGF. Sixteen patients were excluded because they met one or more of the exclusion criteria. Consequently, four patients were enrolled; despite a dose-escalation study, only the initial dose of rh-HGF (0.6 mg/m^2^) was administered. Among the participating subjects, the age was between 40 and 71, and two were male (Table 1). Patients 1, 2 and 4 were diagnosed as having FHSA, and patient 3 as having LOHF. These four patients were not able to receive liver transplantation, because patients 1, 3, and 4 lacked appropriate donors, and patient 2 was over 70 years old. FHSA in patients 1 and 4 was caused by HEV and a supplement containing coenzyme Q-10, respectively, whereas the cause of hepatic failure in patients 2 and 3 was undetermined. Two patients with FHSA (patients 1 and 2) and one with LOHF (patient 3) exhibited hepatic encephalopathy at grade II and V, respectively, whereas the consciousness level of patient 4 with FHSA was not impaired at the time of enrollment. In all patients, markedly prolonged PT and an increase in T-Bil and serum HGF were observed. Patient 2, with FHSA, and patient 3, with LOHF, exhibited reduced liver volume as determined by CT volumetry at enrollment. Treatment with rh-HGF was started between five and seven days after appearance of hepatic encephalopathy. rh-HGF (0.6 mg/m^2^/day) was intravenously administered for 14 days in patients 2 and 4. Patients 1 and 3 required cessation of rh-HGF on days 14 and 13, respectively, because of increased serum creatinine (2.1 mg/dL) and oliguria, respectively. Both of these symptoms were determined to accompany hepatic failure, but not rh-HGF dosing. Thus, these patients were subject to a total of 13- and 12-day HGF administration regimens, respectively. Plasma exchange was performed in all patients. Three patients, except for patient 1 with FHSA caused by HEV, were treated with corticosteroid (Additional file 1, Additional file 2, Additional file 3, Additional file 4). Finally, two of the patients with FHSA (2 and 4) survived, whereas the other two patients died. Patient 1, who had FHSA, died after the study period; patient 3, who had LOHF, died during the study period (Table 1).

**Table 1:** Patient characteristics

**Patient No**.	1	2	3	4
Age/Gender	67/M	71/F	64/F	40/M
Diagnosis/Etiology	FHSA/HEV	FHSA/unknown	LOHF/unknown	FHSA/drug
Reason for not receiving LT	donor^1^	age^2^	donor^1^	donor^1^
Before rh-HGF administration				
Grade of HE	II	II	V	0
Prothrombin time INR (%)	2.07 (33)	1.55 (49)	1.78 (37)	1.62 (43)
Albumin (g/dL)	2.9	3.2	2.9	2.9
T-Bil (mg/dL)	11.2	6.9	11.7	27.6
Direct/total bilirubin ratio	0.58	0.41	0.44	0.71
ALT (IU/L)	32	131	260	253
Serum HGF (ng/mL)	0.77	1.94	1.07	1.88
AFP (ng/ml)	7.0	22.9	3.9	39.7
Liver volume (mL)	1055	595	640	1110
Days between HE and rh-HGF administration (days)	7	5	5	5
Duration of rh-HGF dosing (days)	13	14	12	14
Outcome				
during the study period	alive	alive	dead	alive
during the follow-up period	dead	alive	-	alive

FHSA, fulminant hepatitis subacute type; LOHF, late onset hepatc failure; HEV, hepatitis E virus; LT, liver transplantation; HE, hepatic encephalopathy. ^1^lack of an appropriate donor; ^2^age 70 or over.

### Pharmacokinetics of stepwise infusion of rh-HGF for three hours

**Figure 3:**
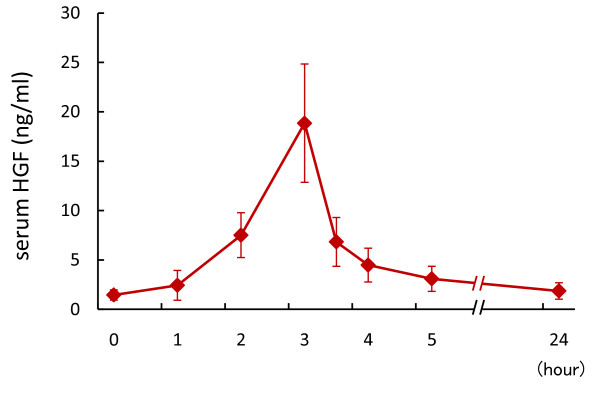
**Sequential changes in serum HGF concentration during and after rh-HGF administration**. rh-HGF (0.6 mg/m^2^) was administered intravenously with a stepwise increase for three hours (0.06 mg/m^2 ^for 60 min, 0.18 mg/m^2 ^for next 60 min, and 0.36 mg/m^2 ^for last 60 min). Serum levels of HGF were measured by ELISA. Sequential changes in (A) serum HGF levels on day 1 of rh-HGF dosing period.

In patients 1, 2, and 3, rh-HGF was administered after plasma exchange. Serum levels of HGF increased in parallel with a stepwise increase of rh-HGF dosing, and reached maximum drug concentration (Cmax) at the end of a three-hour rh-HGF injection (Figure 3). Cmax gradually increased from 18.8 ± 6.0 ng/mL on day 1 to 22.3 ± 9.6 ng/mL on day 11 during the HGF dosing period (Table 2). The mean value of half-life (T_1/2_) was approximately 630 to 840 min. The area under the blood concentration-time curve (AUC) gradually increased, and the clearance (CL) and steady-state volume of distribution (Vdss) appeared to gradually decrease, during the HGF dosing period.

**Table 2:** Pharmacokinetic parameters of rh-HGF

parameters	Estimate values	95% confidence interval	
**Day 1**			
C_max _(ng/mL)	18.8	13.0	24.7
AUC_0-300 _(ng/mL*min)	1485.6	991.3	1979.8
AUC_0-∞ _(ng/mL*min)	1994.0	1214.6	2773.3
T_1/2 _(min)	756.2	526.8	985.7
CL (mL/m^2^/min)	0.000361	0.000160	0.000561
V_dss _(mL/m^2^)	0.125	0.063	0.186

**Day 5**			
C_max _(ng/mL)	21.3	12.8	29.9
AUC_0-300 _(ng/mL*min)	1727.2	1099.7	2354.7
AUC_0-∞ _(ng/mL*min)	2493.8	1647.0	3340.5
T_1/2 _(min)	843.6	540.5	1146.6
CL (mL/m^2^/min)	0.000277	0.000138	0.000416
V_dss _(mL/m^2^)	0.106	0.059	0.153

**Day 11**			
C_max _(ng/mL)	22.3	11.4	33.1
AUC_0-300 _(ng/mL*min)	1965.5	801.6	3129.5
AUC_0-∞ _(ng/mL*min)	3126.4	1355.2	4897.5
T_1/2 _(min)	633.3	318.0	948.6
CL (mL/m^2^/min)	0.000230	0.000095	0.000365
V_dss _(mL/m^2^)	0.088	0.031	0.146

### Intravenous rh-HGF was well tolerated in all patients with FH or LOHF

**Figure 4:**
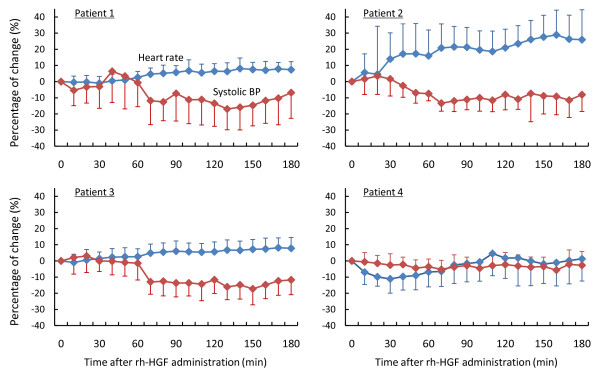
**Blood pressure decreased during infusion of rh-HGF in patients with FH or LOHF**. BP and HR were monitored during rh-HGF infusion for three hours. Intravenous rh-HGF (0.6 mg/m^2^) reduced systolic BP, and increased HR in patients 1, 2 and 3. BP reduction during rh-HGF infusion did not affected patients' general condition. BP immediately recovered following the completion of rh-HGF administration.

Preclinical safety studies revealed that a decrease in BP during rh-HGF infusion and renal toxicity induced by repeated rh-HGF dosing, including an increase in urinary excretion of albumin, were potential adverse events in a human study. In the phase I/II study of patients with FH or LOHF, respiratory status was not affected by rh-HGF administration in any patient, but BP was decreased mildly to moderately from approximately one hour after the beginning of HGF injection in patients 1, 2 and 3 (Figure 4). As HGF reduces BP through dilatation of capacitance vessels, the HR increased up to 30%. However, this decrease in BP did not require cessation of rh-HGF or any vasopressor therapy, and BP returned to resting levels after the completion of HGF administration. Patient 2, who awoke from hepatic encephalopathy on day 3 of the HGF dosing period, did not suffer from any symptoms during HGF administration, even though the HR increased up to ~30% (Figure 4).

All patients showed slight to mild increase in urinary excretion of albumin at enrollment and a decrease in urine volume during the rh-HGF study period. However, repeated doses of rh-HGF did not increase urinary excretion of albumin, and urine volume was affected by several factors other than rh-HGF administration, including volume of infusion, amount of circulating plasma, and diuretic dosing. Although hypokalemia, anemia, a decrease in platelet count, prolonged PT, a decrease in anti-thrombin III, and hematuria were also observed in three of four patients, there was no apparent evidence for a causal relationship between these adverse events and rh-HGF administration. Patient 3, who died of advanced hepatic failure during the observation period, exhibited respiratory failure. However, this severe adverse event was associated with progression of hepatic failure, not rh-HGF; no other severe adverse events directly caused by single or repeated doses of rh-HGF were observed during the study period.

### HGF administration did not show a beneficial effect on hepatic encephalopathy, laboratory data results, or patient survival

Three out of four patients exhibited hepatic encephalopathy at enrollment (Table 1). Patient 1 presented with grade II hepatic encephalopathy at the beginning of protocol therapy. This patient did not recover from hepatic encephalopathy either during or after the study period. The patient ultimately died 68 days after the onset of hepatic encephalopathy (Additional file 1). In patient 2, who had FHSA and ultimately survived, plasma exchange was performed on days 2, 4, and 8 during the HGF dosing period (Additional file 2), and hepatic encephalopathy had improved by day 3. Patient 3 showed advanced hepatic encephalopathy at enrollment. Although the consciousness level was transiently alleviated during the rh-HGF dosing period, hepatic encephalopathy continued to progress during the observation period; the patient died 28 days after the onset of hepatic encephalopathy (Additional file 3). Patient 4 had already recovered from hepatic encephalopathy at enrollment, and did not show any impairment of consciousness level during the study period (Additional file 4). Consequently, we did not observe a definite effect of rh-HGF administration on hepatic encephalopathy.

**Figure 5:**
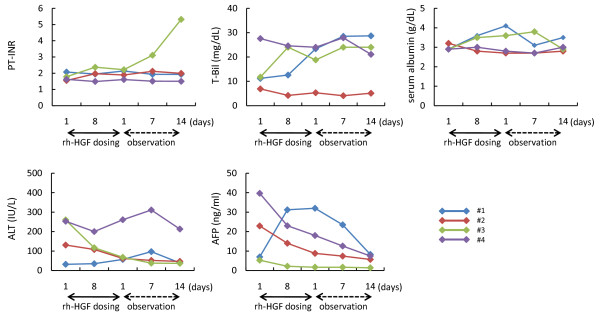
**Changes in laboratory data results during rh-HGF dosing and observation period**. PT-INR, T-Bil, serum albumin, ALT and AFP, were measured before rh-HGF administration (day 1 of rh-HGF dosing); on day 7 of the rh-HGF dosing period; and one, seven and 14 days after the protocol therapy (days 1, 7 and 14 of the observation period, respectively). Laboratory data results were not affected during or after rh-HGF administration.

Laboratory data results, including PT-INR, T-Bil, serum albumin, and ALT, were not affected during the rh-HGF dosing and observation period (Figure 5). In patient 1, serum AFP, which is known to increase not only during development of hepatocellular carcinoma but also liver regeneration, modestly increased during the rh-HGF dosing period, followed by a gradual decrease during the observation period. Conversely, patients 2 and 4, who ultimately survived, exhibited an increase in serum AFP at enrollment, whereas AFP levels gradually decreased throughout the study period. However, no definite effect of rh-HGF dosing on serum AFP levels was observed.

To assess the effect of administration of rh-HGF on patient survival, we selected subjects as a control, who matched each patient in diagnosis (FHSA or LOHF), age (≥45 or <45), gender, PT (<10% or ≥10%), T-Bil (≤18.0 or >18.0 mg/dL) and direct/total bilirubin ratio (≤0.67 or >0.67), from the data of national survey of FH and LOHF in Japan between 1998 and 2006. Consequently, we set 57 control subjects for patients 1 and 2, 13 for patient 3, and 17 for patient 4, and estimated hazard ratios using the stratified proportional hazards model. The survival time from the onset of hepatic encephalopathy or disease in patients treated with rh-HGF was slightly longer than that in control subjects, but the difference was not statistically significant (Table 3).

**Table 3:** Effect of rh-HGF administration on survival time

	hazard ratio	95% CI		*p value*
Survival time from:				
onset of hepatic encephalopathy	0.20	0.03	1.45	0.08
onset of disease	0.28	0.04	2.04	0.18

## Discussion

This clinical trial covered patients with FH, an extremely severe and fatal liver disease: subjects enrolled in this trial are predicted to die without liver transplantation. Indeed, a nationwide survey of the patients with FH or LOHF (1998-2002) in Japan revealed that the survival rate of the patients (n = 192) who met this study's inclusion criteria was 17.7% (n = 34). Additionally, FH is a relatively rare syndrome in Japan (698 patients between 1998 and 2003) [4]; patients with severe complications, especially renal dysfunction and heart failure, were excluded in order to more precisely evaluate the safety and efficacy of the proposed therapy. Therefore, we had difficulty with recruitment of trial subjects. Ultimately, we recruited only four patients to our institute, Kyoto University Hospital, for treatment with the initial dose of rh-HGF.

Predicted adverse events included a decrease in BP, by dilatation of capacitance vessels, and proteinuria. Therefore, we established a stepwise infusion method to avoid a rapid reduction of BP, and confirmed reversibility of renal toxicity through additional preclinical studies. In this clinical trial, rh-HGF was administered intravenously for 12 to 14 days, and severe side effects and complications caused by rh-HGF dosing were not observed. BP was gradually reduced during stepwise infusion of rh-HGF in three of the four patients, whereas repeated doses of rh-HGF did not affect albuminuria. In the first patient, when BP decreased during rh-HGF administration, 200-300 mL of infusion was sufficient to restore BP immediately; prior infusion ameliorated HGF-induced BP reduction, as observed in preclinical animal experiments (Figure 1C). In any event, the decrease in BP observed during HGF infusion was reversible, and did not affect patients' general condition. Although patients 2 and 3, but not 4, also exhibited BP reduction during rh-HGF infusion, their general condition was stable without additional infusion or cessation of rh-HGF. Of particular importance, patient 2, who had awakened from hepatic encephalopathy, showed no symptom or sign during rh-HGF administration. Therefore, we concluded that rh-HGF administered intravenously with a stepwise increase for up to 14 consecutive days was very well tolerated.

In this study, although two of four patients survived, there was no evidence that rh-HGF was effective in improving outcome of patients with FHSA or LOHF. There are three potential reasons for the failure of this trial to demonstrate the efficacy of rh-HGF in patients with FH or LOHF.

First, the dose of rh-HGF and/or the 14-day treatment schedule used in this study might have been too low to produce beneficial effect. The dose chosen for this study was based on a scaling of the doses used in pre-clinical animal studies, and ensured safety in several repeated dose toxicity tests. Also, this dose, corresponding to 0.1 mg/kg in rodents, has been reported to accelerate liver regeneration in normal and partially hepatectomized rats [11]. Conversely, the treatment duration was based on a nationwide survey of FH and LOHF in Japan between 1998 and 2002. In this survey, 90.4% (n = 47) of surviving patients from FHSA and LOHF (n = 52) awaked within 14 days after hepatic encephalopathy occurred, and 71% (n = 135) of non-surviving patients (n = 190) died within 28 days following the onset of hepatic encephalopathy. Therefore, rh-HGF administration for up to 14 days, followed by a 14-day observation period, was considered to be sufficient to evaluate both safety and efficacy. However, in the current study, there was no evidence of inhibited disease progression or stimulated liver regeneration. This suggests either that the dose of rh-HGF administered in this study was insufficient to induce liver regeneration and suppress liver injury, or that the 14-day treatment regimen was too short.

Second, HGF/c-Met pathways may be impaired in patients with FH or LOHF. When rh-HGF was intravenously injected in a bolus, most rh-HGF was distributed into the liver, and development of liver injury or cirrhosis retarded clearance of rh-HGF [23,24]. In this clinical study, serum levels of HGF increased to 10-20 ng/mL (Cmax) just after a stepwise infusion of rh-HGF (0.6 mg/m^2^). HGF is known to stimulate proliferation of both mature hepatocytes and hepatic progenitor cells: less than 10 ng/mL of HGF was sufficient to induce proliferation of primary cultured rat hepatocytes [12,25], and *in vivo *proliferation of rat hepatic progenitor cells was stimulated by serum levels of ~2 ng/mL human HGF [13,26]. In patients with FH, serum levels of growth and growth-inhibitory factors were elevated [27-29], and reciprocal action of these factors in FH patients results in impaired liver regeneration. In this clinical trial, the increase in serum HGF concentration did not lead to improvement of hepatic reserve; furthermore, serum levels of transforming growth factor (TGF)-β, a growth-inhibitory factor, were not affected by HGF administration (Additional file 5). However, patient 1 revealed an increase in serum AFP, a marker of liver regeneration in patients with FH, during rh-HGF dosing period, and gradually decreased after the completion of rh-HGF administration. In contrast, patients 2 and 4, who survived, showed an increase in serum AFP at enrollment, but serum AFP levels decreased during the rh-HGF dosing period. These two patients received PSL in parallel with rh-HGF (Additional files 2 and 4); AFP expression is known to be affected by a glucocorticoid responsive element (GRE) present in the 5'-flanking region of AFP gene [30]. Once serum AFP levels decreased, slowly tapered PSL did not affected serum AFP in these surviving patients. However, AFP expression at enrollment may be suppressed via the GRE, leading to a decrease in serum AFP levels. Therefore, dose escalation or prolonged exposure to rh-HGF may be able to overcome impaired liver regeneration.

Third, both FH and LOHF patients enrolled in this trial were predicted to die without liver transplantation; thus, the subjects already presented with an extremely serious condition. This life-threatening condition was influenced by the degree of impaired hepatic reserve and varying complications. Indeed, in this trial, all eligible patients with FH or LOHF developed hepatic encephalopathy, and the impaired hepatic reserve and general condition varied in severity. In these patients, even though safety could be evaluated, it may be difficult to evaluate the clinical efficacy. Therefore, it will be desirable to examine the clinical efficacy of rh-HGF in additional clinical trials involving patients with less severe conditions.

Systemic administration of potent growth factors could theoretically stimulate premalignant lesions in distant organs. Therefore, in this first clinical trial of rh-HGF, it was prudent to limit systemic therapy to life-threatening conditions. Although the two surviving patients in this study should be observed over the long term, we showed here that repeated doses of intravenous rh-HGF were well tolerated even in patients with a fatal disease. Recent investigations have indicated that HGF has the potential to improve treatment for intractable diseases of various organs, including the nervous system [31,32], lung [33], heart [34-36], intestine [26,37], kidney [38], and vessels [39]. Therefore, the safety assessment of protein-based therapy of HGF described here sheds light on the development of new therapeutic modalities aimed at treating patients with intractable diseases.

## Conclusions

Despite a mild BP reduction during rh-HGF infusion, intravenous rh-HGF at a dose of 0.6 mg/m^2 ^was well tolerated in patients with FH or LOHF. However, there was no evidence that those dose of rh-HGF was effective for the treatment of these patients. Additional studies of rh-HGF at doses higher than 0.6 mg/m^2^, for longer periods, or in treatment of patients with less severe conditions, will be valuable in determining the clinical efficacy of rh-HGF.

## Competing interests

The authors declare no competing interests. Mitsubishi Tanabe Pharma Corporation had no role in the design of the study, in data accrual or analysis, or in preparation of the manuscript.

## Authors' contributions

AI, AM, MN, and IDK conducted preclinical studies. AI, AM, MN, IDK, TM, ST, SH, MY, MF, AS, and HT participated in research design. AI, SH, AS, and HT contributed to preparation of rh-HGF at GMP grade. AI, AM, MN, TM, HM, NY, HS, IDK, TC, and MY provided medical care. ST and MF performed data analysis. AI, AM, MN, ST, AS, and HT wrote or contributed to the writing of the manuscript.

## Supplementary Material

Additional file 1 **Clinical course of patient 1 with FHSA, the first patient receiving intravenous rh-HGF**. We first administered rh-HGF to a 67-year-old Japanese man with FHSA caused by hepatitis E virus infection. On admission, he presented with hepatic encephalopathy, jaundice, ascites, edema, and microhematuria caused by bladder catheter. Although ALT had already decreased to 32 IU/L, we observed thrombocytopenia (6.1 × 10^4^/μL), increased T-Bil (11.2 mg/dL), a marked decrease in serum albumin (2.9 g/dL), and prolonged PT (33%) (PT-INR 2.07), indicating severely impaired hepatic reserve. Serum HGF and AFP levels were 0.77 and 7.0 ng/mL, respectively, and liver volume measured by CT was 1055 mL. Following observation of general condition for two days, administration of rh-HGF (0.6 mg/m^2^/day) was initiated. Because of an increase in serum creatinine level of 2.0 mg/dL, caused by diuretics administration to reduce massive ascites, protocol therapy was discontinued on day 14, resulting in 13-day administration of rh-HGF. Although prolonged PT was stable during rh-HGF dosing and observation period, T-Bil gradually increased and hepatic encephalopathy did not improve. Hepatic failure gradually progressed after the observation period; the patient ultimately died 68 days after the onset of hepatic encephalopathy. PE, plasma exchange; CHDF, continuous hemodiafiltration. Click here for file

Additional file 2 **Clinical course of patient 2 with FHSA, who survived**. The second patient (patient 2) was a 71-year-old Japanese woman with FHSA of undetermined etiology. She presented with mild hepatic encephalopathy with flapping tremor, jaundice, and urinary findings, including proteinuria and microhematuria, caused by bladder catheter. Platelet count and serum albumin level decreased to 6.9 × 10^4^/μL, and 3.2 g/dL, respectively, and PT was prolonged to 49% (PT-INR 1.55). In addition to increased T-Bil level of 6.9 mg/dL, serum ALT level increased to 131 IU/L. Serum HGF and AFP levels were 1.94 and 22.9 ng/mL, respectively, and liver volume was 595 mL. Following observation of general condition for 24 hours, treatment with rh-HGF was initiated, and the protocol therapy was continued for 14 days without any severe adverse events. Hepatic encephalopathy disappeared after plasma exchange (PE) on day 2; consciousness level was not impaired throughout the study period. Intravenous rh-HGF reduced systolic BP. The patients with lucidity, however, did not complain any symptom. Although prednisolone (PSL) was administered to reduce ALT, blood biochemical findings and patient condition were stable throughout the study period. After the completion of the study, biochemical findings were gradually improved, and, finally, the patient survived. Click here for file

Additional file 3 **Clinical course of patient 3, with LOHF, who died within the observation period**. Sixty four-year-old Japanese woman with LOHF of undetermined etiology suffered from advanced hepatic encephalopathy (HE). She presented with platelet count of 9.2 × 10^4^/μL, PT of 37% (PT-INR 1.78), T-Bil level of 11.7 mg/dL, ALT level of 260 IU/L, and serum albumin level of 2.9 g/dL. Serum HGF and AFP levels were 1.07 and 3.9 ng/mL, respectively, and liver volume was 640 mL. Because of oliguria (392 mL/day), protocol therapy was discontinued on day 13, resulting in 12-day rh-HGF dosing. Additionally, PSL was administered to reduce serum ALT, and plasma exchange (PE) and/or continuous hemodiafiltration (CHDF) was performed throughout the study period. Serum ALT levels reduced immediately, and hepatic encephalopathy was transiently improved during rh-HGF dosing period. However, hepatic encephalopathy, prolonged PT, and an increase in T-Bil progressed during the observation period, and the patient died during the observation period (28 days after the onset of hepatic encephalopathy). Click here for file

Additional file 4 **Clinical course of patient 4, with FHSA caused by a drug, who survived**. Forty-year-old Japanese man with FHSA, which was caused by a supplement containing coenzyme Q-10, showed platelet count of 7.0 × 10^4^/μL, PT of 43% (PT-INR 1.62), T-Bil level of 27.6 mf/dL, ALT level of 253 IU/L, and serum albumin level of 2.9 g/dL, but not hepatic encephalopathy (HE), which was temporarily observed before enrollment. Serum HGF and AFP levels were 1.88 and 39.7 ng/mL, respectively, and liver volume was 1110 mL. Administration of rh-HGF was continued for 14 days, and PSL was administered to reduce ALT throughout the study period. An increase in T-Bil and prolonged PT was modestly improved during rh-HGF dosing, followed by further improvement after the observation period. Ultimately, the patient survived. PE; plasma exchange. Click here for file

Additional file 5 **Serum levels of TGF-β were not affected by rh-HGF dosing**. Serum TGF-β concentrations before and after the rh-HGF dosing period were determined by ELISA. Although patient 2 exhibited an increase in serum TGF-β after 14-day rh-HGF administration, there was no significant difference in serum levels of TGF-β (mean ± SE: 230.4 ± 21.0 vs 266.4 ± 68.1 pg/ml, *p = 0.52*). Click here for file
