# Supplementary figures and images for: Safety and pharmacokinetics of recombinant human hepatocyte growth factor (rh-HGF) in patients with fulminant hepatitis: a phase I/II clinical trial, following preclinical studies to ensure safety
Source: J Transl Med. 2011 May 8;9:55. doi: 10.1186/1479-5876-9-55 (PMC3112439; doi:10.1186/1479-5876-9-55)

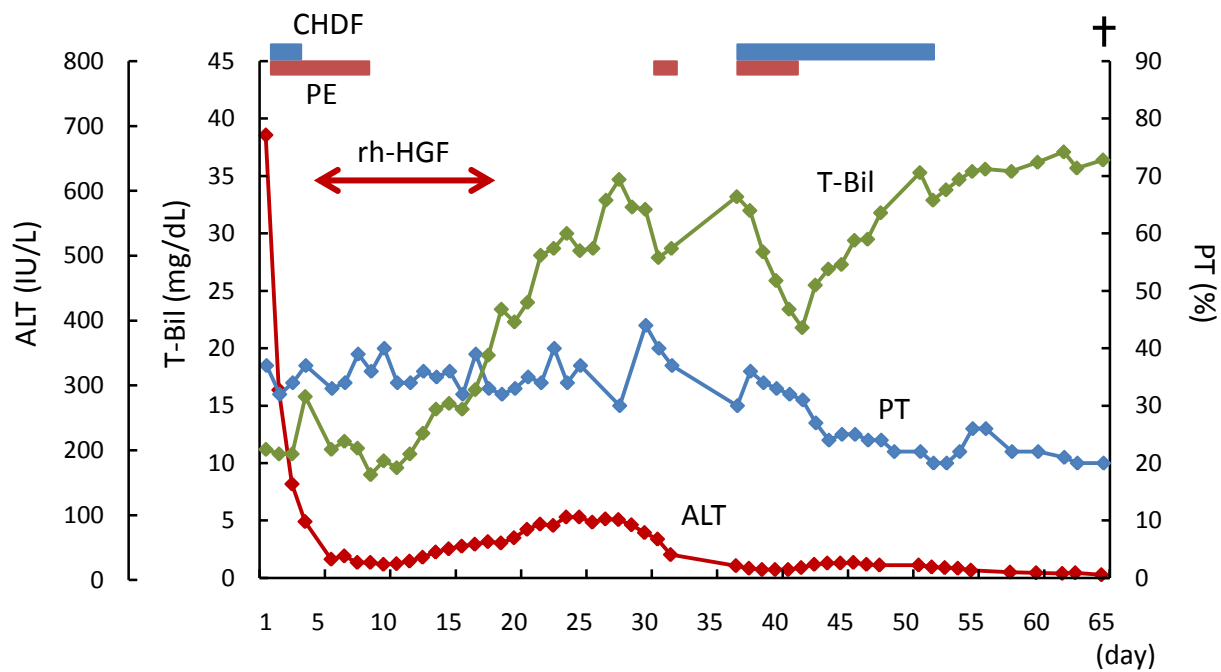

Supplement: Additional file 1 — Clinical course of patient 1 with FHSA, the first patient receiving intravenous rh-HGF. We first administered rh-HGF to a 67-year-old Japanese man with FHSA caused by hepatitis E virus infection. On admission, he presented with hepatic encephalopathy, jaundice, ascites, edema, and microhematuria caused by bladder catheter. Although ALT had already decreased to 32 IU/L, we observed thrombocytopenia (6.1 × 10^4^/μL), increased T-Bil (11.2 mg/dL), a marked decrease in serum albumin (2.9 g/dL), and prolonged PT (33%) (PT-INR 2.07), indicating severely impaired hepatic reserve. Serum HGF and AFP levels were 0.77 and 7.0 ng/mL, respectively, and liver volume measured by CT was 1055 mL. Following observation of general condition for two days, administration of rh-HGF (0.6 mg/m2/day) was initiated. Because of an increase in serum creatinine level of 2.0 mg/dL, caused by diuretics administration to reduce massive ascites, protocol therapy was discontinued on day 14, resulting in 13-day administration of rh-HGF. Although prolonged PT was stable during rh-HGF dosing and observation period, T-Bil gradually increased and hepatic encephalopathy did not improve. Hepatic failure gradually progressed after the observation period; the patient ultimately died 68 days after the onset of hepatic encephalopathy. PE, plasma exchange; CHDF, continuous hemodiafiltration. [file 1479-5876-9-55-S1.PDF]

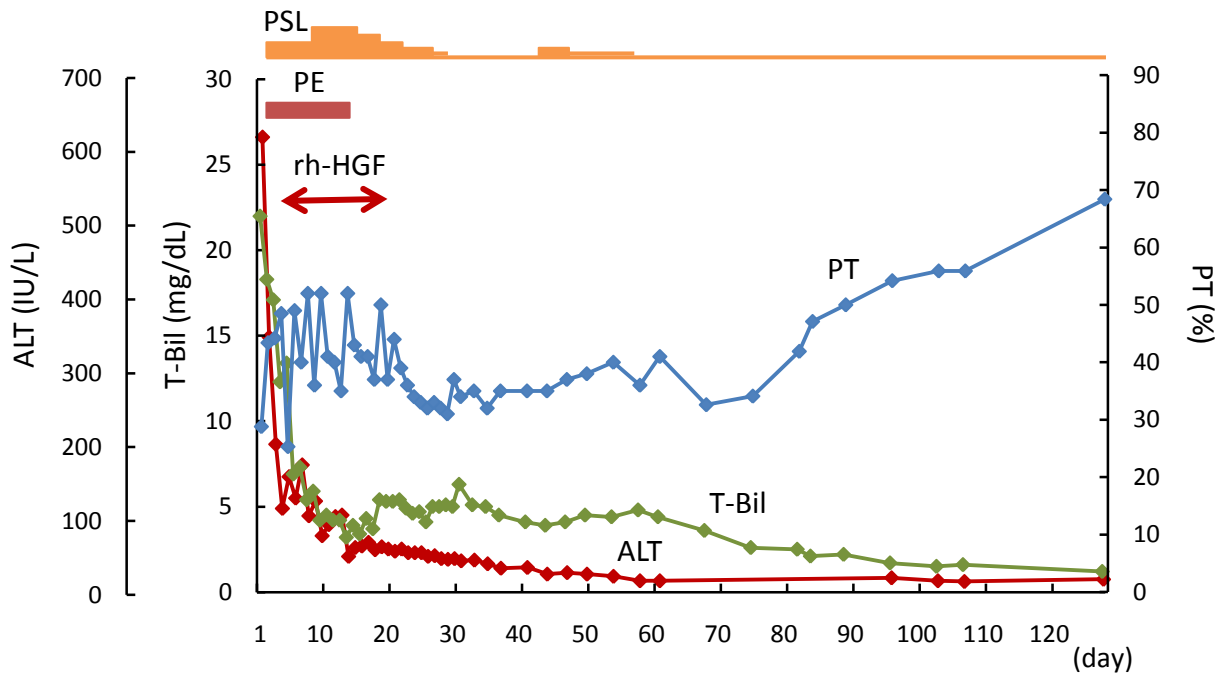

Supplement: Additional file 2 — Clinical course of patient 2 with FHSA, who survived. The second patient (patient 2) was a 71-year-old Japanese woman with FHSA of undetermined etiology. She presented with mild hepatic encephalopathy with flapping tremor, jaundice, and urinary findings, including proteinuria and microhematuria, caused by bladder catheter. Platelet count and serum albumin level decreased to 6.9 × 10^4^/μL, and 3.2 g/dL, respectively, and PT was prolonged to 49% (PT-INR 1.55). In addition to increased T-Bil level of 6.9 mg/dL, serum ALT level increased to 131 IU/L. Serum HGF and AFP levels were 1.94 and 22.9 ng/mL, respectively, and liver volume was 595 mL. Following observation of general condition for 24 hours, treatment with rh-HGF was initiated, and the protocol therapy was continued for 14 days without any severe adverse events. Hepatic encephalopathy disappeared after plasma exchange (PE) on day 2; consciousness level was not impaired throughout the study period. Intravenous rh-HGF reduced systolic BP. The patients with lucidity, however, did not complain any symptom. Although prednisolone (PSL) was administered to reduce ALT, blood biochemical findings and patient condition were stable throughout the study period. After the completion of the study, biochemical findings were gradually improved, and, finally, the patient survived. [file 1479-5876-9-55-S2.PDF]

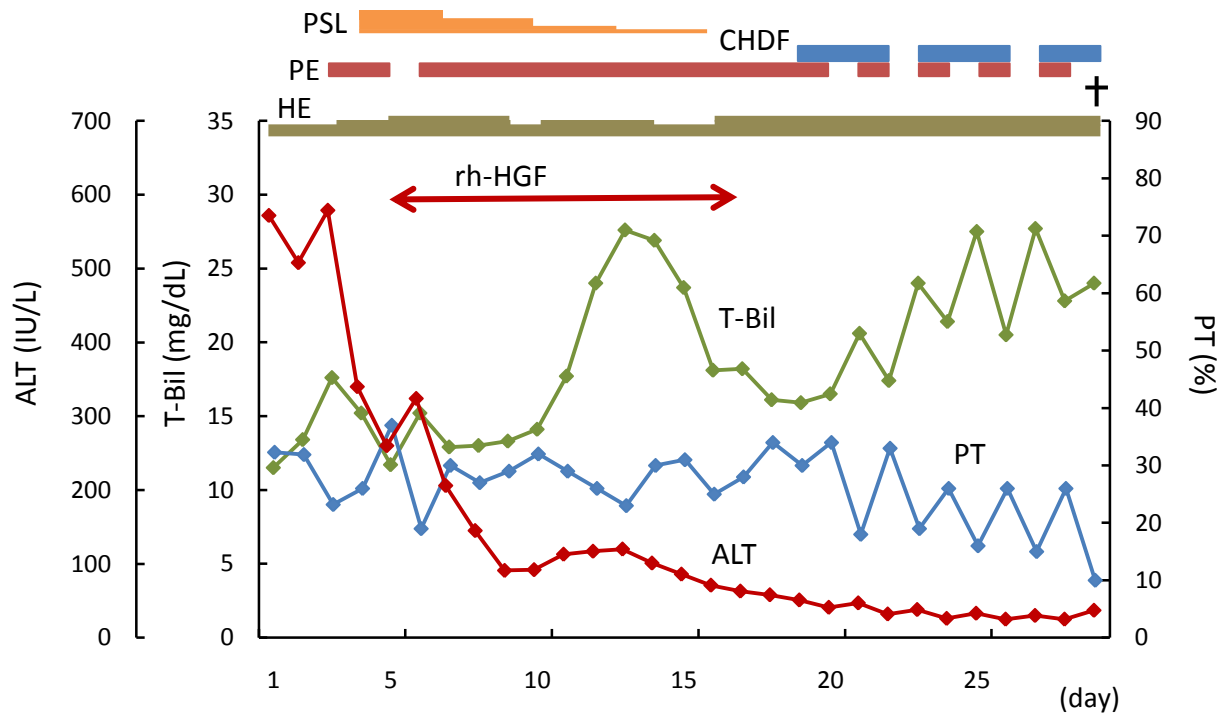

Supplement: Additional file 3 — Clinical course of patient 3, with LOHF, who died within the observation period. Sixty four-year-old Japanese woman with LOHF of undetermined etiology suffered from advanced hepatic encephalopathy (HE). She presented with platelet count of 9.2 × 10^4^/μL, PT of 37% (PT-INR 1.78), T-Bil level of 11.7 mg/dL, ALT level of 260 IU/L, and serum albumin level of 2.9 g/dL. Serum HGF and AFP levels were 1.07 and 3.9 ng/mL, respectively, and liver volume was 640 mL. Because of oliguria (392 mL/day), protocol therapy was discontinued on day 13, resulting in 12-day rh-HGF dosing. Additionally, PSL was administered to reduce serum ALT, and plasma exchange (PE) and/or continuous hemodiafiltration (CHDF) was performed throughout the study period. Serum ALT levels reduced immediately, and hepatic encephalopathy was transiently improved during rh-HGF dosing period. However, hepatic encephalopathy, prolonged PT, and an increase in T-Bil progressed during the observation period, and the patient died during the observation period (28 days after the onset of hepatic encephalopathy). [file 1479-5876-9-55-S3.PDF]

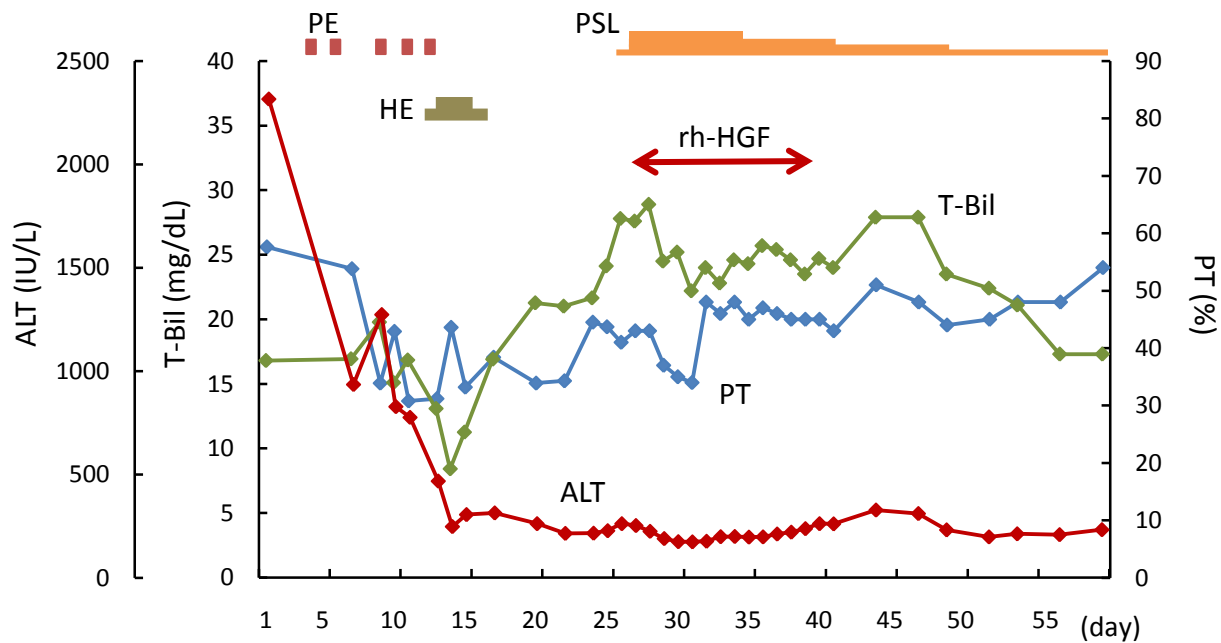

Supplement: Additional file 4 — Clinical course of patient 4, with FHSA caused by a drug, who survived. Forty-year-old Japanese man with FHSA, which was caused by a supplement containing coenzyme Q-10, showed platelet count of 7.0 × 10^4^/μL, PT of 43% (PT-INR 1.62), T-Bil level of 27.6 mf/dL, ALT level of 253 IU/L, and serum albumin level of 2.9 g/dL, but not hepatic encephalopathy (HE), which was temporarily observed before enrollment. Serum HGF and AFP levels were 1.88 and 39.7 ng/mL, respectively, and liver volume was 1110 mL. Administration of rh-HGF was continued for 14 days, and PSL was administered to reduce ALT throughout the study period. An increase in T-Bil and prolonged PT was modestly improved during rh-HGF dosing, followed by further improvement after the observation period. Ultimately, the patient survived. PE; plasma exchange. [file 1479-5876-9-55-S4.PDF]

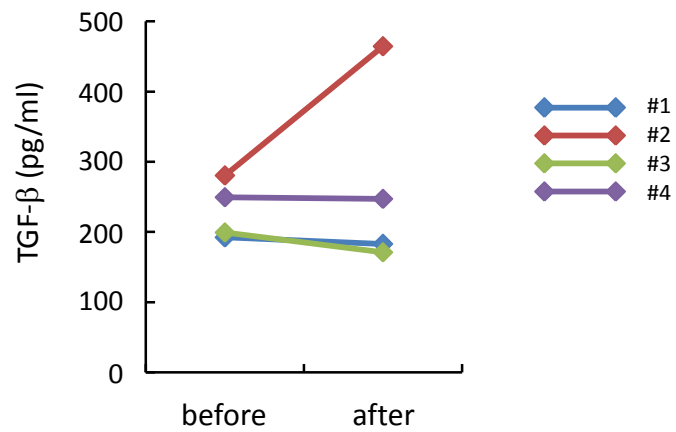

Supplement: Additional file 5 — Serum levels of TGF-β were not affected by rh-HGF dosing. Serum TGF-β concentrations before and after the rh-HGF dosing period were determined by ELISA. Although patient 2 exhibited an increase in serum TGF-β after 14-day rh-HGF administration, there was no significant difference in serum levels of TGF-β (mean ± SE: 230.4 ± 21.0 vs 266.4 ± 68.1 pg/ml, p = 0.52). [file 1479-5876-9-55-S5.PDF]
